# P04.35. Sustained effects of a mindfulness-based classroom intervention on behavior in urban, underserved children

**DOI:** 10.1186/1472-6882-12-S1-P305

**Published:** 2012-06-12

**Authors:** M Klatt, E Browne, K Harpster, J Case-Smith

**Affiliations:** 1 The Ohio State University College of Medicine, Columbus, USA

## Purpose

To investigate the initial and sustained effects of Move-Into-Learning (MIL), an 8-week Mindfulness-Based Intervention (MBI), delivered in the classroom, that utilized yoga movement, music, written and visual arts, designed to reduce stress and improve behavior in at-risk elementary students.

## Methods

MIL was implemented in a low income, urban neighborhood with 3rd grade students (n=41) in a school under academic emergency with many behavior problems. A pre to post test single group design with a 2 month follow up measure was used to investigate the behavioral changes in the children. The MIL program utilized a standardized protocol consisting of mindfulness meditation, yoga movement/breathing in harmony with music, and appreciative inquiry (AI) exercises that required students to express themselves in the written and visual arts. Students were evaluated by their classroom teacher pre/post MIL intervention using the Connors Behavior Rating Scale, identifying problem behaviors that had occurred in the month prior to the assessments.

## Results

Children in the intervention group showed significant improvement in hyperactivity (F [1,40]=10.18; p= .002), and highly significant differences in the ADHD index (F[1,40]= 27.0; p<.001), and cognitive/inattentiveness (F [1,40]=35.50; p<.001) subscales with medium to large effect sizes. In the two month post-intervention measure (n=20), the ADHD index, and hyperactivity continued to improve between the post intervention and the two month follow up, while cognitive/inattentive behaviors (F(1,19)=8.56; p=.01) significantly improved.

## Conclusion

Teachers, administrators, and parents may all recognize a child whose behavior is negatively impacted by stress, but they may not be familiar with programs that can effectively provide strategies for stress reduction. MIL is one such program, providing research to practice evidence of effective stress reduction, feasible for classroom delivery, with outcome data supporting improved behavior for at-risk children, with effects sustained beyond the intervention.

